# Screening of an FDA-Approved Drug Library: Menadione Induces Multiple Forms of Programmed Cell Death in Colorectal Cancer Cells via MAPK8 Cascades

**DOI:** 10.3390/ph18081145

**Published:** 2025-07-31

**Authors:** Liyuan Cao, Weiwei Song, Jinli Sun, Yang Ge, Wei Mu, Lei Li

**Affiliations:** 1 Institute for Translational Medicine on Cell Fate and Disease, Shanghai Ninth People’s Hospital, Shanghai Jiao Tong University School of Medicine, Shanghai 200025, China; caoliyuan@sjtu.edu.cn; 2 Center for Single-Cell Omics, School of Public Health, Shanghai Jiao Tong University School of Medicine, Shanghai 200025, China; a20020926sww@163.com (W.S.); sunjinli1989@shsmu.edu.cn (J.S.); geyang1026@icloud.com (Y.G.); 3 State Key Laboratory of Molecular Oncology, National Cancer Center/National Clinical Research Center for Cancer/Cancer Hospital, Chinese Academy of Medical Sciences and Peking Union Medical College, Beijing 100021, China; 4 Department of Critical Care Medicine, Ruijin Hospital, Shanghai Jiao Tong University School of Medicine, Shanghai 200025, China

**Keywords:** drug screening, menadione, colorectal cancer, programmed cell death, necroptosis, MAPK8

## Abstract

**Background**: Colorectal cancer (CRC) is a prevalent gastrointestinal malignancy, ranking third in incidence and second in cancer-related mortality. Despite therapeutic advances, challenges such as chemotherapy toxicity and drug resistance persist. Thus, there is an urgent need for novel CRC treatments. However, developing new drugs is time-consuming and resource-intensive. As a more efficient approach, drug repurposing offers a promising alternative for discovering new therapies. **Methods**: In this study, we screened 1068 small molecular compounds from an FDA-approved drug library in CRC cells. Menadione was selected for further study based on its activity profile. Mechanistic analysis included a cell death pathway PCR array, differential gene expression, enrichment, and network analysis. Gene expressions were validated by RT-qPCR. **Results**: We identified menadione as a potent anti-tumor drug. Menadione induced three programmed cell death (PCD) signaling pathways: necroptosis, apoptosis, and autophagy. Furthermore, we found that the anti-tumor effect induced by menadione in CRC cells was mediated through a key gene: *MAPK8*. **Conclusions**: By employing methods of cell biology, molecular biology, and bioinformatics, we conclude that menadione can induce multiple forms of PCD in CRC cells by activating MAPK8, providing a foundation for repurposing the “new use” of the “old drug” menadione in CRC treatment.

## 1. Introduction

CRC is one of the most common malignant cancers, which ranks third in cancer morbidity and second in cancer mortality [1]. Although the majority of CRC patients are 65 years and older, there is an increase in the number of diagnosed CRC patients under the age of 50, which is probably related to modern lifestyle and environmental factors [1,2]. CRC can be further divided into colon cancer and rectal cancer according to lesion site, with symptoms such as bloody stools, diarrhea, and local abdominal pain. According to pathological classification, CRC can be divided into adenocarcinoma, adenosquamous carcinoma, and undifferentiated carcinoma [3]. According to research data, the five-year overall survival rates of CRC patients are closely related to the TNM stage: stage I, 74%; stage IIA, 66%; stage IIB, 58%; stage IIC, 37%; stage IIIA, 73%; stage IIIB, 46%; stage IIIC, 28%; and stage IV, 5% [4]. This survival crossover is attributed to the high local aggressiveness of T4b tumors (tumors invading adjacent organs) in stage IIC. The treatment of CRC primarily relies on surgery, supplemented by pharmacotherapy [5]. For patients at a late stage who have missed the surgical window, pharmacotherapy is the only choice, which includes chemotherapy, targeted therapy, and immunotherapy. Chemotherapeutic agents, including 5-fluorouracil, oxaliplatin, and capecitabine, remain the backbone of CRC treatment, often in combination with targeted therapies such as bevacizumab (monoclonal antibody of VEGF) [6,7]. Immunotherapy has also emerged as a promising strategy for CRC treatment, with an increasing number of FDA-approved immunotherapeutic agents being adopted clinically, including immune checkpoint inhibitors targeting programmed cell death ligand 1 (PD-L1) [8]. Recently, nanomedicine-based drug delivery systems (e.g., polymeric nanoparticles [9] and liposomes [10]) have been developed to improve targeting efficacy and reduce drug toxicity [11]. Several comprehensive reviews have documented the current landscape of therapeutic approaches [6] and delivery strategies [11] in CRC. Despite recent progress in CRC drug development, several limitations persist, such as the toxic side effects of chemotherapy drugs, the limitations of targeted therapy due to *KRAS/NRAS/BRAF* gene mutations, and the drug resistance of tumor cells caused by long-term medication [7,12]. Therefore, investigating and developing new drugs has become an urgent need for clinical treatment.

PCD refers to an active death process of a cell that occurs after receiving certain signals or external stimuli in order to maintain the stability of the internal environment [13]. Unlike passive and disordered cell death caused by external adverse factors such as radiation, toxins, and pathogens, PCD is an active and organized way of cell death that is regulated by relevant signaling pathways and is reversible. Moreover, PCD occurs in not only abnormal physiological states or diseases but also the normal development process of an individual [14]. Traditional theory suggests that cell death can be divided into apoptosis and necrosis, among which only apoptosis is regulated by cell signaling pathways. Apoptotic cells have typical morphological and biochemical changes, including cell shrinkage, chromatin concentration, apoptotic bodies, DNA fragmentation, and protein degradation [15], and its mechanism is caspase-dependent or caspase-independent based on whether caspase is involved [16]. However, in recent years, several other forms of PCD, such as autophagy and necroptosis, have also been discovered. Autophagy refers to a process of cells using lysosomes to degrade self-components, including cytoplasm and organelles and it can be divided into three types: macroautophagy, microautophagy, and chaperone-mediated autophagy [17]. Autophagy is a double-edged sword of cancer; on the one hand, autophagy inhibits cancer cell proliferation, and on the other hand, autophagy is a stress mode adopted by cancer cells when challenged by drug therapy, which is related to cancer resistance [18]. Therefore, regulating autophagy is also a potential strategy for anti-tumor therapy. Necroptosis is a novel form of cell death that was first reported by Degterev et al. in 2005 [19]. Necroptosis has similar morphological characteristics to traditional necrosis, but researchers found that necroptosis is mediated by specific signal pathways (e.g., RIPK1/RIPK3/MLKL signal), which are ordered and regulatable [20]. Previously, the studies of necroptosis mainly focused on inflammatory diseases (e.g., systemic inflammatory response syndrome [21]); however, in recent years, the potential role of necroptosis in tumor development and therapy has gained attention. For example, research proved that RIPK3 was downregulated in CRC [22], breast cancer [23], melanoma [24], and leukemia [25]. Therefore, inducing cell death of cancer cells by activating/regulating PCD (such as necroptosis, apoptosis, and autophagy) is a promising strategy for cancer therapy.

Despite the urgent need for effective and groundbreaking anti-tumor drugs, candidate drugs must undergo comprehensive assessments and careful considerations before any clinical or pre-clinical trials are carried out, owing to significant human efforts and financial costs. In order to save medical resources, “new uses for old drugs” has become another strategy for the development of “new drugs”. “Old drugs” refers to drugs that have been marketed or are undergoing clinical trials, while ”new uses” refers to the discovery of new indications or new uses of a drug in clinical treatment [26]. Therefore, repurposing old drugs is regarded as a rapid and efficient strategy for drug development, offering advantages of cost savings and shorter research cycles compared to traditional methods. Additionally, the well-documented toxicity as well as side effects of old drugs make them safer and more reliable options. A prime example is the application of aspirin in the prevention and treatment of cardiovascular diseases. Following this strategy, we screened 1068 “old drugs” in an FDA-approved drug library in order to find a drug with anti-tumor ability for “new uses” in CRC treatment, and menadione is the best candidate, which is also known as vitamin K3, usually be used to prevent vitamin K deficiency caused by long-term oral antibiotics. In our study, we demonstrated that menadione activates MAPK8, inducing various forms of PCD in CRC cells, including necroptosis, apoptosis, and autophagy. This underscores the potential of repurposing menadione for treating CRC, providing a new strategy for clinical application.

## 2. Results

### 2.1. Screening of an FDA-Approved Drug Library to Identify Effective Compounds of Inducing Cell Necroptosis

**Figure 1:**
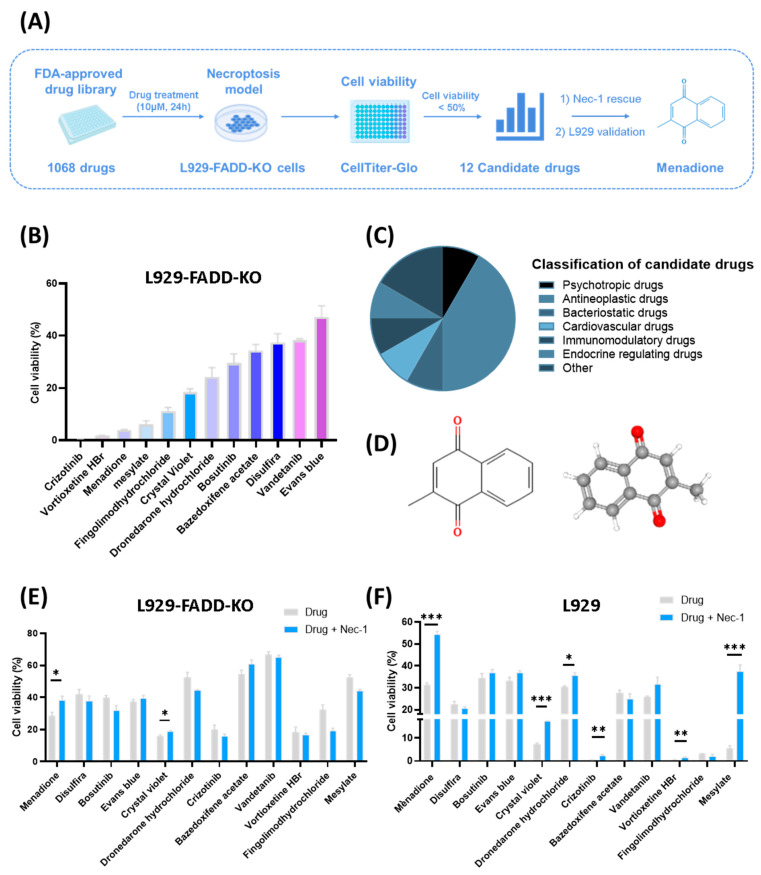
Screening of an FDA-approved drug library to identify effective compounds of inducing cell necroptosis. (**A**) Flow chart of drug screening to identify potential anti-tumor drugs that could induce necroptotic cell death. 1068 drugs in an FDA-approved drug library were screened, and menadione was identified as a promising candidate. (**B**) Cell viability of L929-FADD-KO cells after treating with 12 candidate drugs (10 μM, 24 h) that caused >50% reduction in cell viability (n = 3). (**C**) Classification of 12 candidate drugs. (**D**) Chemical structure of menadione. (**E**,**F**) Nec-1 (30 μM) was used to rescue cell death induced by candidate drugs (10 μM, 24 h) in L929-FADD-KO cells (**E**) and wild-type L929 (**F**). Statistical significance was evaluated using unpaired two-tailed Student’s *t*-test. Data of cell viability are presented as mean ± standard error of the mean (SEM, n = 3). * *p* < 0.05, ** *p* < 0.01, *** *p* < 0.001.

Currently, many anti-tumor therapies induce tumor cell death by promoting cell apoptosis. However, tumor cells often evade apoptosis, leading to drug resistance and chemotherapy failure. Necroptosis is also a form of PCD that is activated in a caspase-independent manner, offering new insights for overcoming drug resistance in cancer treatment. In order to identify compounds that can effectively induce necroptosis, we started out by screening potential drugs that could induce necroptotic cell death (Figure 1A). To identify such compounds, a necroptosis-sensitive cell line, L929-FADD-KO, was selected to screen 1068 drugs in an FDA-approved drug library (TargetMol, L4200), which has been widely adopted in high-throughput screening studies for drug repurposing [27,28,29]. After treating with drugs (10 μM) for 24 h, cell viability was detected using the CellTilter-Glo kit. Tumor necrosis factor alpha (TNF-α), a well-characterized inducer of PCD, was used as a positive control. The assay system responded robustly to TNF-α, and the cytotoxic effects could be effectively rescued by the necroptosis inhibitor Necrostatin-1 (Nec-1) (Supplementary Figure S1), thereby confirming the specificity and functionality of our screening conditions.

After preliminary screening, there were 12 drugs that caused >50% reduction in cell viability, with varying degrees of cytotoxicity (Figure 1B, Supplementary Table S1). According to current clinical functions, these 12 drugs were divided into psychotropic drugs, antineoplastic drugs, bacteriostatic drugs, cardiovascular drugs, immunomodulatory drugs, endocrine-regulating drugs, and other drugs (Figure 1C, Table 1). To precisely identify drugs that induced necroptosis, we used Nec-1 to rescue cell death caused by these drugs. Among the drugs that achieved improved cell viability after Nec-1 application in L929-FADD-KO cells, menadione (*p* = 0.034) and crystal violet (*p* = 0.049) proved to be potential necroptosis inducers (Figure 1E, Supplementary Table S2). In the wild-type L929 cell line for validation, menadione (*p* < 0.001) and crystal violet (*p* < 0.001) were also rescued by Nec-1 (Figure 1F, Supplementary Table S3). Considering drug toxicity and rescue results in L929-FADD-KO/wild-type L929, we finally selected menadione, also known as vitamin K3, as the best candidate drug. The structure of menadione is presented in Figure 1D, and its function was investigated in the following study.

### 2.2. Menadione Exerted Strong Anti-Tumor Effects on Colorectal Cancer Cells

**Figure 2:**
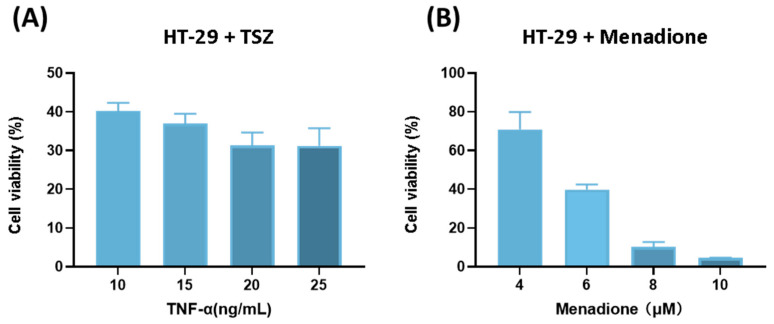

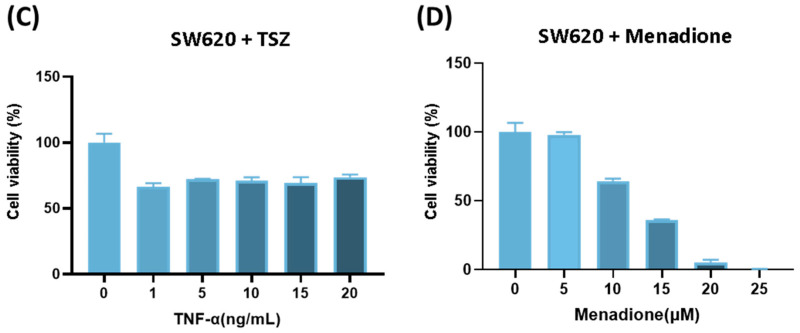
Menadione exerted strong anti-tumor effects on colorectal cancer cell lines. (**A**) Different concentrations of TNF-α were administered to HT-29 cells combined with Smac mimetics (100 nM) and Z-VAD-FMK (20 μM). Cell viability was assessed after 24 h (n = 3). (**B**) Different concentrations of menadione were administered to HT-29 cells and cell viability was assessed after 24 h (n = 3). (**C**) Different concentrations of TNF-α were administered to SW620 cells combined with Smac mimetics (100 nM) and Z-VAD-FMK (20 μM). Cell viability was assessed after 24 h(n = 3). (**D**) Different concentrations of menadione were administered to SW620 cells and cell viability was assessed after 24 h (n = 3). S: Smac mimetics; Z: Z-VAD-FMK. Data of cell viability are presented as mean ± SEM (n = 3).

After identifying menadione as a potent inducer of necroptotic cell death through screening, we investigated its ability to kill CRC cells for potential clinical applications. Previous experiments have demonstrated that menadione could induce necroptosis; therefore, we selected TNF-α as a positive control. According to related studies, Smac mimetics and ubiquitin caspase inhibitor Z-VAD-FMK can cause necroptosis in CRC cell line HT-29 cells, and their combined application with TNF-α enhances the capacity to induce necroptosis in HT-29 cells, which is a classic model for studying necroptosis [30]. Therefore, we first validated the effects of Smac mimetics (birinapant was used in this study) and Z-VAD-FMK on HT-29 cells, as shown in Supplementary Figure S2A, which was consistent with previous studies. Subsequently, Smac metrics and Z-VAD-FMK were combined with TNF- α (which is referred to as TSZ in the following contents) (Supplementary Figure S2B). The ability of TNF-α to induce cell death increased with increasing concentration (Figure 2A). Next, we explored the cytotoxic effects of menadione on HT-29. The results showed that menadione effectively induced CRC cell death, and its anti-tumor ability increased with increasing concentration and a high concentration of menadione could almost kill all HT-29 cells (Figure 2B). We further investigated the effects of TSZ and menadione on another CRC cell line, SW620. Surprisingly, menadione exerted much more potent anti-tumor ability on SW620 compared with TSZ (Figure 2C,D). These results implicated that menadione exerted a strong anti-tumor effects on CRC cells, which is probably more significant than TNF-α, with great potential for further exploration and application.

### 2.3. Menadione Induced Not Only Necroptosis, but Also Apoptosis and Autophagy in Colorectal Cancer Cells

**Figure 3:**
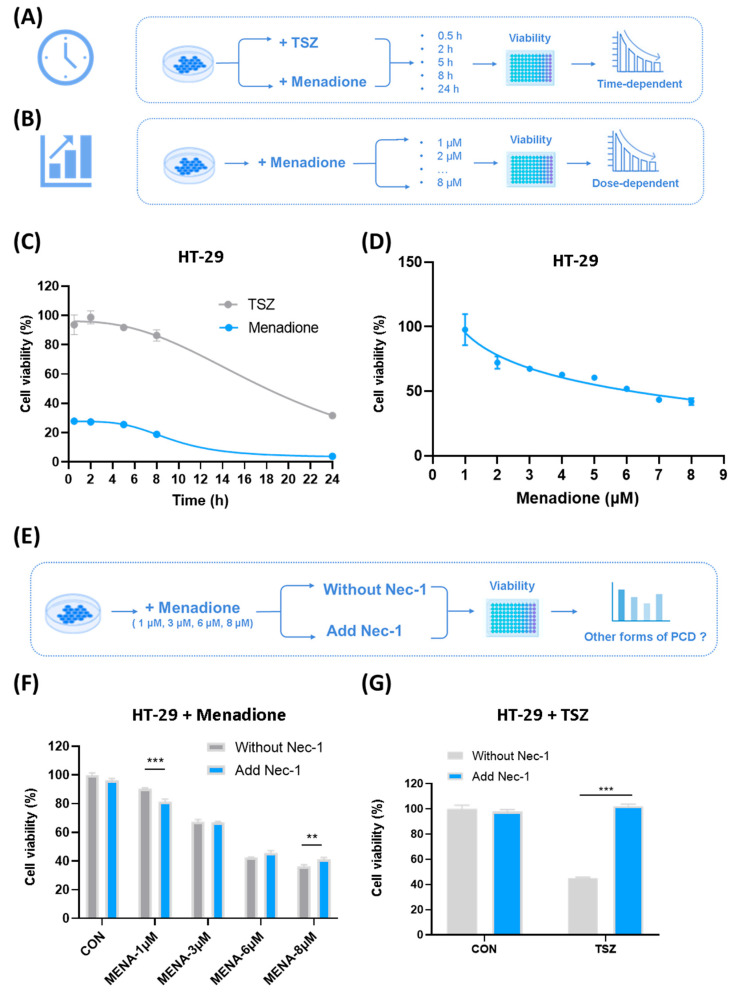
Necroptosis was not the only form of cell death induced by menadione in colorectal cancer cells. (**A**,**B**) Flow charts of time-course and concentration–gradient experiments. (**C**) Menadione (8 μM) or TSZ (T: TNF-α, 10 ng/mL; S: Smac mimetics, 100 nM; Z: Z-VAD-FMK, 20 μM) was administered to HT-29 cells and cell viability was assessed at indicated time points (0.5 h, 2 h, 5 h, 8 h, and 24 h) (n = 3). Some error bars too small to display clearly. (**D**) Menadione of different doses was added to HT-29 cells and cell viability was determined 1 h after treatment (n = 3). Some error bars too small to display clearly. (**E**) Flow chart of Nec-1 rescue assay. (**F**) Menadione (1 μM, 3 μM, 6 μM and 8 μM) was administered to HT-29 cells with or without Nec-1 (10 μM) and cell viability was assessed after 1 h (n = 3) (**G**) TSZ (T: TNF-α, 10 ng/mL; S: Smac mimetics, 100 nM; Z: Z-VAD-FMK, 20 μM) was administered to HT-29 cells with or without Nec-1 (10 μM) and cell viability was assessed after 24 h (n = 3). Statistical significance was evaluated using an unpaired two-tailed Student’s *t*-test. Data of cell viability are presented as mean ± SEM (n = 3). ** *p* < 0.01, *** *p* < 0.001.

To comprehensively investigate the time-dependent and dose-dependent effects of menadione on CRC cells, we conducted a series of time-course and concentration-gradient experiments (Figure 3A–D). The results showed that menadione rapidly exerted its tumoricidal effects within 1 h after administration, and the cytotoxic effects reached stability after 24 h (Figure 3A,C). Moreover, compared with TSZ, menadione exhibited much stronger cytotoxic effects on CRC cells, which was almost 3 times (Figure 3C). Considering dose effects of menadione on CRC cells, we carried out a concentration-gradient experiment to identify the optimal concentration for subsequent experiments (Figure 3B,D). The results showed that the anti-tumor effect of menadione on HT-29 increased with increasing concentration, and the optimal concentration range for menadione was from 3 μM to 8 μM (cell viability was approximately 40–70%) (Figure 3D). We also performed additional experiments using the non-cancerous human cell line HEK293T alongside other CRC cell lines, and the results showed that menadione induced significantly less cytotoxicity in HEK293T cells compared to CRC cell lines, suggesting a degree of selectivity (Supplementary Figure S3). In order to further validate the cell death form induced by menadione, we used Nec-1 to rescue necroptotic cell death (Figure 3E–G). Although Nec-1 could alleviate the cell death induced by 8 μM menadione (Figure 3F), the rescue effect was much less significant than that on TSZ (Figure 3G), indicating that apart from necroptosis, other forms of cell death also participated. Moreover, Nec-1 not only failed to rescue the cell death induced by 1 μM menadione but even increased cell death rate (Figure 3F). We speculated that this phenomenon was probably because different concentrations of menadione activated different the cell death signalings in HT-29. According to previous studies, low doses of certain drugs often cause cell apoptosis, while high doses further induce necroptosis [31,32]. Therefore, we inferred that when 1 μM menadione was applied to HT-29, the major form of cell death was apoptosis, and Nec-1 could promote cell apoptosis under certain conditions [33]; while when 8 μM menadione was applied, the necroptosis pathway was activated and participated in cell death, thus Nec-1 alleviated cell death.

**Figure 4:**
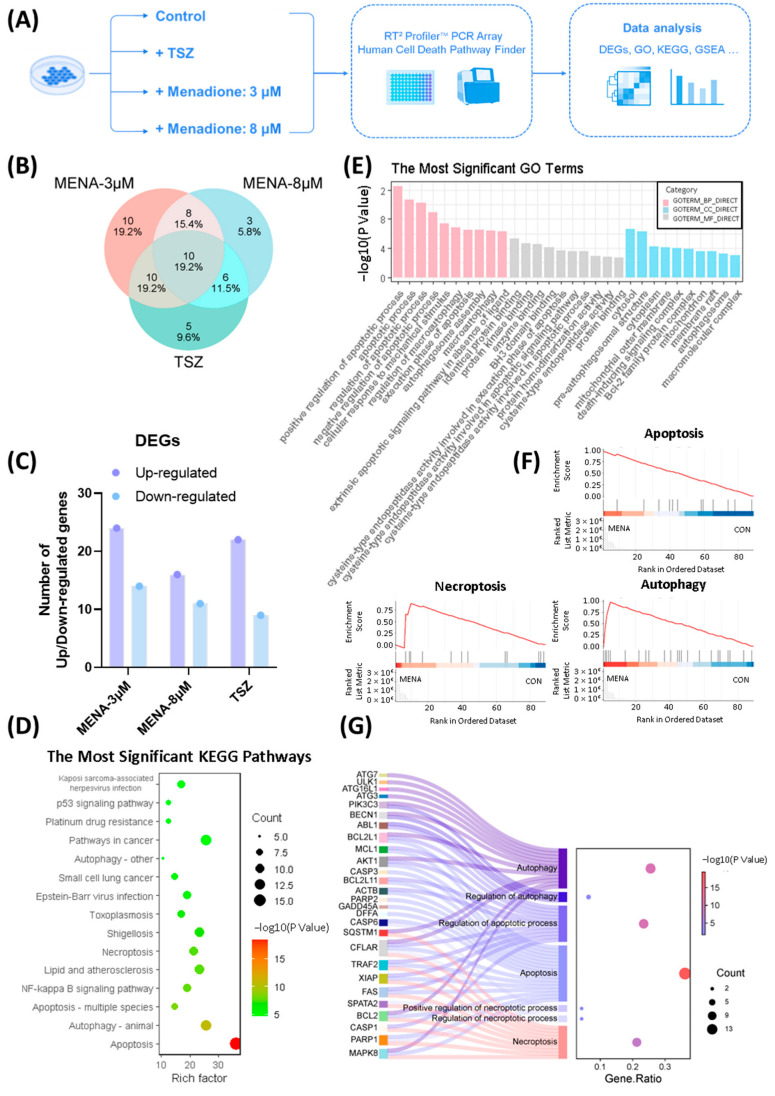
Menadione induced not only necroptosis, but also apoptosis and autophagy in colorectal cancer cells. (**A**) Flow chart of PCR Array assay and study design. (**B**,**C**) Differential analysis was performed and DEGs of experimental groups (MENA-3 μM, MENA-8 μM, and TSZ) were summarized in Venn diagram (**B**) and histogram (**C**). (**D**,**E**) Enrichment analysis was performed on menadione groups, including KEGG pathway analysis (**D**) and GO enrichment analysis (**E**). The most significant KEGG pathways (Top 15) were summarized in (**D**) and the most significant GO pathways (Top 10 of BP, CC, and MF) were summarized in (**E**). (**F**) Representative GSEA pathways: necroptosis, apoptosis and autophagy. GSEA was performed based on the average log_2_ FC of MENA-3 μM and MENA-8 μM treatment groups relative to the control group, to identify pathways consistently regulated across both conditions. (**G**) Interactive analysis of necroptosis, apoptosis and autophagy pathways. The Sankey diagram on the left displays genes (e.g., *ATG7*, *CFLAR*, *BCL2*, and *MAPK8*) mapped to biological processes of necroptosis, apoptosis and autophagy. The bubble plot on the right indicates enrichment degree and statistical significance of each pathway.

Given that necroptosis was not the only form of cell death induced by menadione, we used RT^2^ Profiler™ PCR Array Human Cell Death Pathway Finder for a full investigation of underlying mechanisms (Figure 4A). Apart from the control group, there were three other groups: 3 μM menadione (MENA-3 μM), 8 μM menadione (MENA-8 μM), and TSZ. Fold change (FC) was calculated by using the ∆∆CT method, and a gene with |Log2 FC| > 1 was regarded as a differentially expressed gene (DEG) (genes with Log2 FC >1 were upregulated genes, and genes with Log2 FC < −1 were downregulated genes). There were 38 DEGs in the MENA-3 μM group (24 upregulated and 14 downregulated), 27 DEGs in the MENA-8 μM group (16 upregulated and 11 downregulated), and 31 DEGs in the TSZ group (22 upregulated and 9 downregulated). The results are shown in Table 2 and Figure 4B,C. Given that death signaling induced by menadione in HT-29 was related to but different from that of TNF, KEGG (Kyoto encyclopedia of genes and genomes) and GO (Gene ontology) analysis was carried out on DEGs of menadione groups (Figure 4D,E), and Enrichment Map of Cytoscape software (version: 3.7.2) was applied to further investigate the interactions and interconnections among different pathways (Supplementary Figure S4). The results showed that the necroptosis pathway was among the most significant KEGG pathways (Figure 4D). In addition, apoptosis and autophagy pathways were also activated remarkably (Figure 4D,E). Apart from analysis on DEGs, Gene set enrichment analysis (GSEA) was carried out for comprehensive analysis of all genes. The results showed that necroptosis, apoptosis, and autophagy pathways were up-regulated in menadione groups (Figure 4F). Moreover, DEGs and pathways associated with necroptosis, apoptosis, and autophagy were further analyzed, and the result was summarized in Figure 4G, illustrating the relationships between indicated genes and pathways. This figure showed that there were intersections among genes underlying the mechanism of menadione-induced necroptosis, apoptosis, and autophagy (Figure 4G). We demonstrated that menadione induced not only necroptosis but also apoptosis and autophagy in CRC cells, with these mechanisms being closely interconnected and finely regulated.

### 2.4. The Anti-Tumor Effect Induced by Menadione in Colorectal Cancer Cells Was Mediated Through MAPK8

**Figure 5:**
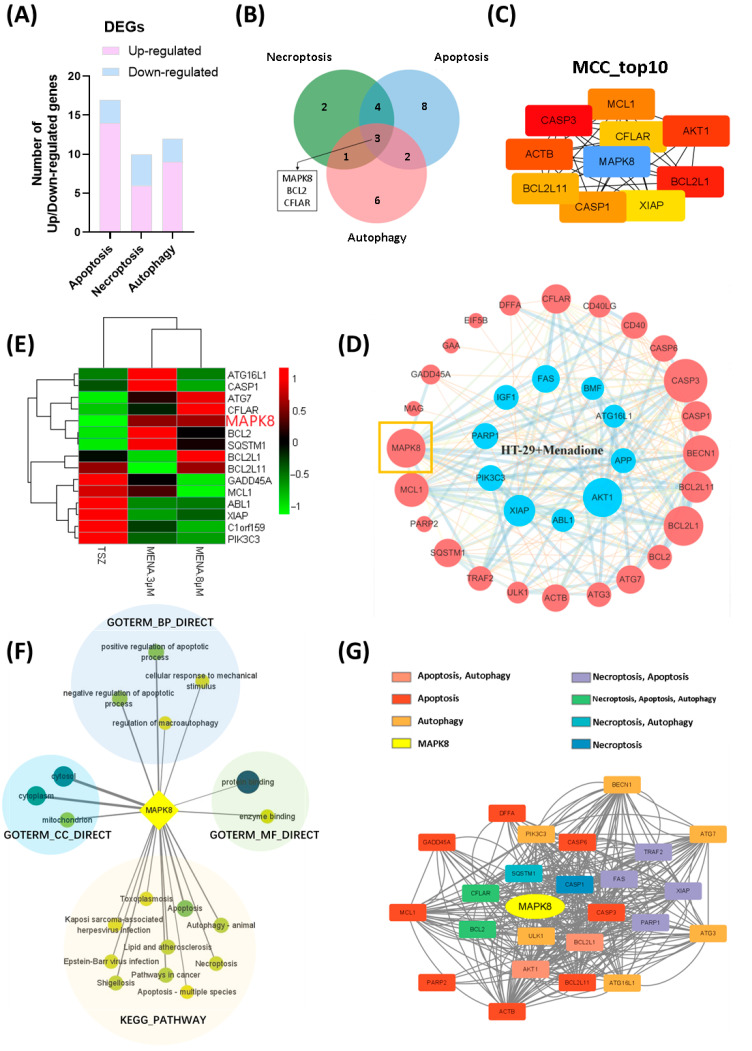
The anti-tumor effect induced by menadione in colorectal cancer cells was mediated through MAPK8. (**A**) Number of up/down-regulated DEGs in pathways of apoptosis, necroptosis, and autophagy after menadione treatment. (**B**) Venn diagram of DEGs in pathways of apoptosis, necroptosis, and autophagy after menadione treatment. (**C**) Core hub genes of menadione groups. Cytohubba was used to predict hub genes of 47 DEGs. The MCC algorithm was applied, and top 10 hub genes were displayed. (**D**) PPI network of DEGs after menadione treatment, visualized by Cytoscape. The nodes in red represent up-regulated DEGs after menadione treatment, and nodes in blue indicate down-regulated DEGs. The node size represents the extent of the gene’s interaction with other genes, with larger nodes indicating a greater number of interacting genes. The edge thickness represents the combined score between two genes, with thicker lines indicating a higher score. The boxed region emphasizes MAPK8 as a core gene in the network. (**E**) Heatmap of DEGs among menadione and TSZ groups. The color of the heatmap squares represents gene expression level, with darker colors indicating a higher gene expression level. (**F**) GO and KEGG pathways involving the hub gene MAPK8. Node size and node color represent the number of enriched DEGs in each pathway, with larger sizes and darker colors indicating a higher number of DEGs. (**G**) PPI network of DEGs that participate in necroptosis, apoptosis, and autophagy pathways. The node color represents the pathways each gene participates in, and the edge indicates protein interaction between two genes.

To further investigate the key regulator of menadione-induced PCD in CRC cells, we applied KEGG Mapper to explore detailed signaling transduction of DEGs in the necroptosis, apoptosis, and autophagy pathway regulation (Supplementary Figure S5A–C). KEGG Mapper showed 10 DEGs in the necroptosis pathway (6 upregulated and 4 downregulated), 17 DEGs in the apoptosis pathway (14 upregulated and 3 downregulated), and 12 DEGs in the autophagy pathway (9 upregulated and 3 downregulated) (Figure 5A, Table 3). The Venn diagram showed three DEGs among the intersection of necroptosis, apoptosis, and autophagy pathways: *MAPK8*, *BCL2*, and *CFLAR* (Figure 5B). To explore molecule interaction and core regulating genes, we used the STRING database for analysis of protein–protein interaction (PPI), and the result showed that DEGs of menadione groups shared a complex network of protein interaction and molecule interconnection (Supplementary Figure S6). Next, Cytohubba, a plug-in of Cytoscape, was used to predict hub genes of the PPI network, and *MAPK8* turned out to be a core hub gene (Figure 5C). *MAPK8* was an upregulated DEG in the menadione-treated group. In PPI network analysis, *MAPK8* shared a high degree of interaction with other genes (Figure 5D), indicating that MAPK8 was a key regulating molecule. In addition, compared with the TSZ-treated group, there was no significant change in the expression of *MAPK8* in the TSZ group (the Log2 FC was only −1.2), suggesting that probably MAPK8 was the key molecule involved in the unique mechanism of menadione, which was different from TNF-α (Figure 5E). Focusing on MAPK8, we found that MAPK8 participated in multiple pathways that were enriched in GO and KEGG analysis, especially cell-death-related pathways, for example, Necroptosis, Apoptosis, Apoptosis-multiple species, and Autophagy—animal (Figure 5F, Supplementary Figure S7A,B). Pathways involving MAPK8 were summarized in Figure 5F. Given that MAPK8 was closely related to PCD pathways, PPI network analysis was applied on DEGs participating in necroptosis, apoptosis, and autophagy, and the result showed that MAPK8 shared a wide interaction network with other DEGs (Figure 5G). These results indicated that MAPK8 was a key regulator in menadione-induced death signaling and probably was a potential target in cancer therapy.

### 2.5. Validation of Programmed Cell Death and Upregulation of MAPK8 Cascades Induced by Menadione in Colorectal Cancer Cells

**Figure 6:**
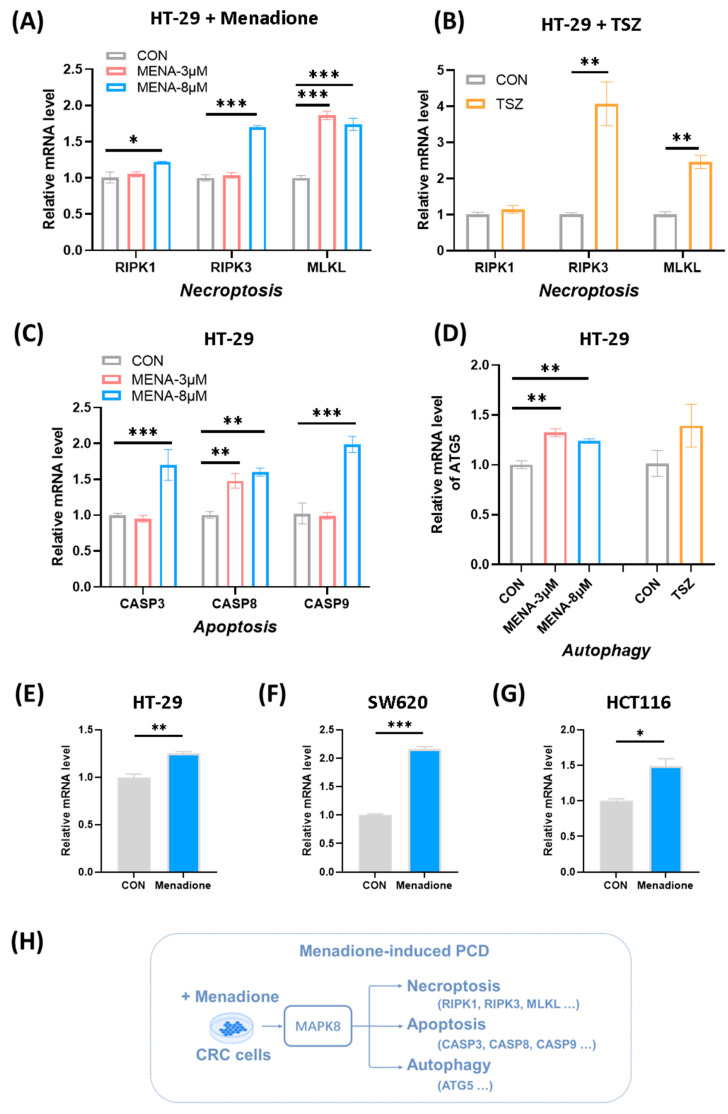
Validation of programmed cell death and upregulation of MAPK8 cascades induced by menadione in colorectal cancer cells (**A**–**D**). Relative transcriptional expression level of PCD-related genes (necroptosis, apoptosis, and autophagy) after menadione application was measured by RT-qPCR (n = 3). Menadione: 3 μM or 8 μM; TSZ: TNF-α, 10 ng/mL; Smac mimetics, 100 nM; Z-VAD-FMK, 20 μM. (**E**–**G**) The relative mRNA levels of MAPK8 were measured by RT-qPCR following treatment with menadione (8 μM) in HT-29, SW620, and HCT116 cells (n = 3). (**H**) A schematic diagram of the mechanism by which menadione induces multiple forms of PCD. Data are presented as mean ± SEM (n = 3). * *p* < 0.05, ** *p* < 0.01, *** *p* < 0.001.

For further validation of PCD induced by menadione, the expression level of PCD-related genes was measured by RT-qPCR. As expected, menadione application increased the expression of RIPK1, RIPK3, and MLKL, indicating necroptosis activation (Figure 6A), similar to the effects observed with TSZ (Figure 6B). Additionally, menadione activated the expression of genes in apoptosis (Figure 6C) and autophagy pathways (Figure 6D). These results confirmed that menadione induced not only necroptosis but also apoptosis and autophagy in CRC cells. Moreover, the expression of MAPK8 was measured, and the results suggested that menadione increased MAPK8 expression in several CRC cell lines (Figure 6E–G). This further validated the significant role of MAPK8 in menadione induced cell death. Taken together, we speculated that when menadione was applied to CRC cells, it activated multiple forms of PCD, including pathways of necroptosis, apoptosis, and autophagy, and the underlying mechanism was regulated through MAPK8 (Figure 6H).

### 2.6. Drug Targets of Menadione and Role of MAPK8 in CRC

**Figure 7:**
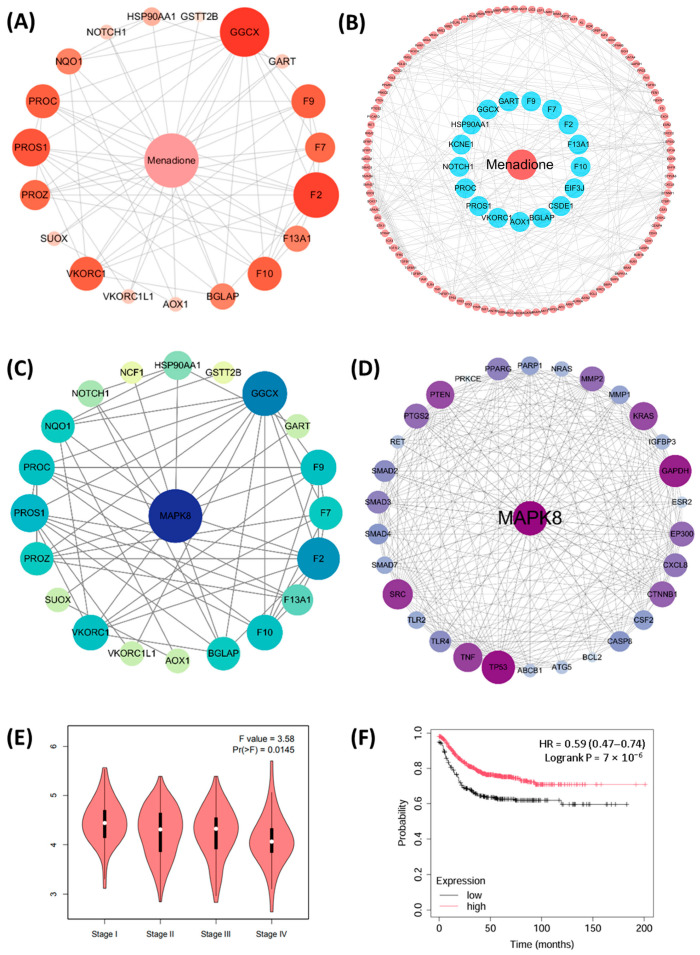
Drug targets of Menadione and the role of MAPK8 in CRC. (**A**) PPI analysis of the 33 drug targets was performed using the STRING database, setting a threshold of PPI score > 0.400. Visualization was performed using Cytoscape, where node size and color intensity represent the degree of interaction with other genes. Larger nodes and darker colors indicate a higher number of interacting genes. (**B**) The DisGeNET database was utilized to retrieve disease targets associated with CRC (search term: colorectal cancer, C1527249), selecting those with a score > 0.3, resulting in a total of 264 targets. A PPI analysis was performed between these 264 CRC-related genes and 33 drug targets of menadione, with a threshold set at PPI score > 0.950. Visualization was performed using Cytoscape, where blue nodes represent the drug targets of menadione and red nodes the CRC disease targets. (**C**) PPI analysis of MAPK8 and menadione drug targets was conducted using the STRING database, with a threshold set as PPI score > 0.400. Visualization was performed using Cytoscape software, where the size and color intensity of the nodes indicate the degree of interactions with other genes. Larger nodes and darker colors reflect a greater number of interacting genes. (**D**) Analysis of MAPK8 in conjunction with 264 CRC-related genes was performed using the STRING database, with a threshold set as PPI score > 0.700. This analysis identified 30 genes that exhibit interactions with MAPK8. Visualization was performed using Cytoscape, where the size and color intensity of the nodes represent the degree of interactions with other genes. Larger nodes and darker colors indicate a greater number of interacting genes. (**E**) Expression level of MAPK8 in CRC patients at different stages (GEPIA database). (**F**) Kaplan–Meier survival analysis of MAPK8 (229664_at) expression in CRC patients. Patients were stratified into high-expression (n = 852) and low-expression (n = 315) groups based on the optimal cutoff value automatically determined by the Kaplan–Meier Plotter tool.

We further utilized the Pharmmapper database to predict potential drug targets of menadione and identified 18 drug targets (Table 4), whose pharmacophore structures are shown in Supplementary Figure S8. In addition, the databases Drugbank and Therapeutic Target Database (TTD) were also applied to predict drug targets of menadione; the results are summarized in Table 5 with a total of 33 drug targets. We conducted the PPI analysis of the 33 drug targets using the STRING database, with results visualized by Cytoscape (Figure 7A). Next, utilizing the DisGeNET database, CRC-associated genes were identified. The interaction of these 264 CRC-associated genes and their targeting for menadione were displayed (Figure 7B). Given that MAPK8 is acting as the core regulator, we further investigated the relationship between MAPK8 and menadione drug targets; the result showed that MAPK8 indeed shared a close interaction network with menadione targets (Figure 7C). The potential intercorrelation of MAPK8 and CRC-related genes was also analyzed and visualized (Figure 7D). Finally, the clinical databases were applied for analysis of MAPK8 in CRC patients. Results show that the expression of MAPK8 decreases with disease progression of CRC patients (Figure 7E), and MAPK8 expression is closely associated with patient survival (Figure 7F). Collectively, these findings suggest that MAPK8 not only plays a central role in the interaction network of menadione targets and CRC-associated genes but also holds clinical relevance as a potential target in CRC.

## 3. Discussion

Starting out with an FDA-approved drug library, our research group used a necroptosis-sensitive cell line L929-FADD-KO, for screening of 1068 drugs, and the results showed that menadione could effectively induce PCD, with great potential to be a potential anti-cancer drug. Next, we applied menadione to human CRC cell lines in order to investigate its anti-tumor ability. In fact, cell toxicity experiments confirmed our hypothesis that menadione could indeed induce cancer cell death, and high concentrations of menadione could kill almost all CRC cells. However, we found that Nec-1, a specific inhibitor of necroptosis, failed to rescue the CRC cell death caused by menadione, which was beyond expectation. This implied that, apart from necroptosis, there were also other forms of cell death caused by menadione, which could not be alleviated by Nec-1. Although Nec-1 alleviated the cell death caused by 8 μM menadione, it aggravated cell death induced by 1 μM menadione. According to previous studies, low doses of certain drugs often cause cell apoptosis, while high doses further cause necroptosis [31,32]. Therefore, we inferred that when 1 μM menadione was applied to HT-29, the major form of cell death was apoptosis, and Nec-1 could promote cell apoptosis under certain conditions [33]; while when 8 μM menadione was applied, the necroptosis pathway was activated and participated in cell death regulation, thus Nec-1 alleviated cell death.

Due to unexpected results of the Nec-1 rescue assay, we applied the Cell Death PCR Array to investigate cell death induced by menadione and underlying mechanisms. Results showed that menadione activated pathways of necroptosis, apoptosis, and autophagy. In fact, these three forms all belong to PCD and interconnect with each other. Apoptosis and necroptosis are closely related; for example, the classic TNF signaling pathway involves both apoptosis and necroptosis. TNF-α binds to tumor necrosis factor receptor 1 (TNFR1) and activates multiple pathways, including nuclear factor kappa B (NF-κB), RIPK1-independent apoptosis (RIA), RIPK1-dependent apoptosis (RDA), and necroptosis [20]. TNF-α binds to TNFR1 and recruits downstream molecules including RIPK1, TNFR1-associated death domain protein (TRADD), adapter protein TRAF2/5, and E3 ubiquitin ligase cIAP 1/2. If TRADD subsequently recruits caspase-8 and fas-associated via death domain (FADD), RIA signaling is activated. If RIPK1 undergoes dimerization and combines with caspase-8 and FADD, RDA is induced [20]. When caspase-8 activity is inhibited, the RIPK1 dimer binds to RIPK3 to form the RIPK1-RIPK3 complex (also known as the necrosome), which further recruits and phosphorylates MLKL. MLKL, the executor of necroptotic cell death, ultimately leads to necroptosis [20]. Therefore, it is evident that necroptosis shares the same upstream pathway with apoptosis, and studies have shown that necroptosis and apoptosis can switch to each other under certain conditions [34,35]. Apoptosis and autophagy also interrelate with each other. Autophagy could promote cell apoptosis or protect cells from apoptosis, depending on metabolic status, nutrient supply, and stimuli received by cells [36,37]. Similar is the relationship between autophagy and necroptosis. Autophagy could serve as a cell protective mechanism to inhibit necroptosis or promote necroptosis under some conditions [38]. From above, it is obvious that menadione could activate several forms of cell death, including necroptosis, apoptosis, and autophagy, which are mediated via a complex regulatory network.

Confirming that menadione activated necroptosis, apoptosis, and autophagy in CRC cells, we next figured out that MAPK8 was the key regulator, which was upregulated after menadione treatment, and the bioinformatics analysis indicated that MAPK8 was a core molecule in the regulatory network. Moreover, the expression of MAPK8 did not significantly change in the TSZ group, suggesting that probably MAPK8 was the key molecule involved in the unique mechanism of menadione, which was different from TNF-α. MAPK8 (mitogen-activated protein kinase 8), also known as JNK1, is a member of the MAPK family. The MAPK family is a group of serine/threonine kinases that play important regulating roles in many physiological and pathological processes [39]. As a member of the MAPK family, MAPK8 can activate and phosphorylate transcription factors (such as AP-1), thereby activating downstream genes and playing an important role in cell death, proliferation, differentiation, and inflammatory responses [40]. It has been found that sustained activation of MAPK8 is closely related to cell apoptosis, and it has been confirmed that MAPK8 is a pro-apoptotic molecule that can activate pro-apoptotic genes like BAX and BAD, inhibit the activity of anti-apoptotic proteins BCL-2 and BCL2L1, and promote the induction of apoptosis [41]. MAPK8 also plays a regulating role in the autophagy signaling pathway. MAPK8 activates the expression of autophagy-related genes such as ATG5, ATG7, and BECN1 by phosphorylating its downstream transcription factor AP-1 [42]. In the necroptosis pathway, the phosphorylation and activation of RIP3K subsequently activates MAPK via MLKL, along with Ca^2+^-dependent mechanisms [42]. The application of JNK inhibitors can alleviate cell necroptosis, inhibit mitochondrial depolarization, and reduce ROS generation [42].

Further analysis of MAPK8 in CRC patients revealed that the expression of MAPK8 decreased with disease progression, and patients with low MAPK8 expression exhibited significantly shorter overall survival. Low expression of MAPK8 may be an adverse factor for disease progression, and therefore, MAPK8 may become a target for the treatment of advanced CRC patients. These analyses indicate that MAPK8 is an important target, menadione is a promising drug to induce PCD, and our study provided a novel clinical strategy for treating CRC patients.

## 4. Materials and Methods

### 4.1. Cell Culture

L929 and CRC cell lines (HT-29, SW620) were obtained from the American Type Culture Collection (ATCC, Manassas, VA, USA). L929 and SW620 cells were cultured in DMEM (BasalMedia, Shanghai, China; Cat# L110KJ), supplemented with 10% FBS (Excel, Shanghai, China; Cat# FSD500) and 1% penicillin streptomycin (BasalMedia; Cat# S110JV). HT-29 cells were cultured in McCoy’s 5A Medium (BasalMedia; Cat# L630KJ), supplemented with 10% FBS and 1% penicillin streptomycin. All the cell lines were maintained in the cell incubator (37 °C, 5% CO_2_) and passaged to appropriate density (usually 1:3) every 2–3 days. Specifically, cells were seeded at a density of 10,000 cells per well in 96-well plates for cell viability assays and 6.0 × 10^5^ cells per well in 6-well plates for RNA extraction, depending on the assay requirements.

### 4.2. Drug Treatment and Cell Viability Assay

For drug treatment, cells were digested and cultured at appropriate density one day in advance, and cells were treated with drugs after they were completely attached to the plate. During the drug screening process, all compounds from the drug library (TargetMol, Boston, MA, USA; Cat# L4200) were used at a concentration of 10 μM. For the necroptosis rescue assay, Nec-1 (TargetMol; Cat# T1847) was used at a concentration of 30 μM. Smac mimetics/birinapant (TargetMol; Cat# T6007) was used at a concentration of 100 nM. Z-VAD-FMK (TargetMol; Cat# T6013) was used at a concentration of 20 μM. Menadione was dissolved in dimethyl sulfoxide (DMSO) prior to use in all in vitro experiments, and the final concentration of DMSO in all treatments did not exceed 0.1% (*v*/*v*), a level that is commonly reported to have minimal effects on cell viability. The concentration of menadione used in this study varied from 1 μM to 10 μM, according to individual assay, and the concentration of TNF-α varied from 1 ng/mL to 25 ng/mL. The treatment time of the drug was mainly 24 h, and specific experiments may involve adjustments. Cell viability was detected after drug treatment using the CellTiter-Glo^®^ Luminescent kit (Promega, Madison, WI, USA; Cat# G7570) according to protocol. This kit is based on a homogeneous method to determine the number of viable cells in culture according to quantitation of the ATP present.

### 4.3. mRNA Isolation and Quantitative PCR

The mRNA from colon cancer cells was isolated using the RNeasy Mini Kit (QIAGEN, Hilden, Germany; Cat# 74104). The extracted mRNA was then reverse transcribed into cDNA utilizing the PrimeScript™ RT Reagent Kit with gDNA Eraser (Takara, Shiga, Japan; Cat# RR047A). For real-time quantitative PCR (RT-qPCR), the 2x SYBR Green qPCR Master Mix (Bimake, Houston, TX, USA; Cat# B21202) was employed, and fluorescence detection was performed using the Bio-Rad CFX96 Touch Real-Time PCR Detection System (Bio-Rad, Hercules, CA, USA). The RT-qPCR primers used in this study are as follows: *BAX*-F: AGGGTTTCATCCAGGATCGAGCAG; *BAX*-R: ATCTTCTTCCAGATGGTGAGCGAG; *ATG5*-F: GCAACTCTGGATGGGATTGC; *ATG5*-R: AGGTCTTTCAGTCGTTGTCTGAT; *RIPK1*-F: TATCCCAGTGCCTGAGACCAAC; *RIPK1*-R: GTAGGCTCCAATCTGAATG-CCAG; *RIPK3*-F: TGGCCCCAGAACTGTTTGTT; *RIPK3*-R: CGGTTGGCAACTCAAC-TTCTC; *MLKL*-F: GGAAGTGTCGCAGCATTCTC; *MLKL*-R: ACCGCCTCCTGAG-GGAAA; *MAPK8*-F: CTGAAGCAGAAGCTCCACCA; *MAPK8*-R: CACCTAAAGGAGAGGGCTGC; β-Actin-F: ACTACCTCATGAAGATCCTCA; β-Actin-R: CAGGAGGAGCAATGATCTTGA.

### 4.4. PCR Array and Data Processing

For investigation of genes involved in cell death-related pathways, we applied RT^2^ Profiler™ PCR Array Human Cell Death Pathway Finder (QIAGEN; Cat# 330231). Reverse transcription was performed using the RT2 First Strand Kit (QIAGEN; Cat# 330401), and PCR array was carried out according to kit protocols. The fold change (FC) was calculated using the following formula: ΔCt = Ct (Gene of Interest) − Ct (Reference Gene); ΔΔCt = ΔCt (Test Group) − ΔCt (Control Group); FC = 1/(2(−ΔΔCt)).

### 4.5. Bioinformatics Analysis

For differential analysis, genes with |Log2 FC| > 1 were regarded as differentially expressed genes (DEGs). Genes with Log2 FC > 1 were upregulated genes, and genes with Log2 FC < −1 were downregulated genes. The DAVID database (URL: https://david.ncifcrf.gov/; version: v2024q2) [accessed on 15 December 2024] was used to perform enrichment analysis of DEGs, including GO enrichment analysis (BP, CC, and MF) and KEGG pathway enrichment analysis. The results were exported, and visualizations were generated using the *ggplot2* package in R studio (version: 4.1.0) [12]. The STRING database [10] (URL: https://string-db.org; version: 12.0) [accessed on 15 August 2023] was used to analyze the protein–protein interaction (PPI) of DEGs, menadione drug targets, and CRC disease targets. The software Cytoscape [11] (version: 3.7.2) was used for visualization, generating the PPI network map showing interactions between the genes. The plugin of Cytoscape, Enrichment Map [13], was applied to visualize the interaction networks of GO and KEGG enrichment results, producing interaction network maps.

## 5. Conclusions

In summary, our study demonstrates that menadione, an FDA-approved compound, exerts potent anti-tumor effects in CRC cells by simultaneously inducing necroptosis, apoptosis, and autophagy, which is mediated through activation of MAPK8. These findings highlight the potential of drug repurposing as a cost-effective and time-saving strategy for CRC treatment. However, the current study is limited to in vitro assays, and further validation in animal models and clinical settings will provide additional evidence for the therapeutic potential of menadione. Future work should also explore the combinatorial potential of menadione with existing therapies and investigate its therapeutic efficacy in CRC.

## Figures and Tables

**Table 1:** Classification of 12 candidate drugs.

Classification	Drugs
Psychotropic drugs	Vortioxetine HBr
Antineoplastic drugs	Menadione, Bosutinib, Crizotinib, Vandetanib, Mesylate
Bacteriostatic drugs	Crystal Violet
Cardiovascular drugs	Dronedarone hydrochloride
Immunomodulatory drugs	Fingolimodhydrochloride
Endocrine regulating drugs	Bazedoxifene acetate
Other	Disulfira, Evans blue

**Table 2:** DEGs of experimental groups.

Menadione-3μM			Menadione-8μM			TSZ		
Gene Symbol	Change	Log2FC	Gene Symbol	Change	Log2FC	Gene Symbol	Change	Log2FC
*CASP1*	up	17.39877	*BCL2L1*	up	9.977538	*CASP9*	up	11.03449
*ATP6V1G2*	up	12.31594	*ULK1*	up	8.808996	*CD40LG*	up	10.80574
*CD40*	up	10.82597	*DEFB1*	up	8.258613	*GADD45A*	up	10.51166
*DPYSL4*	up	8.68331	*BCL2L11*	up	8.228723	*BCL2L11*	up	8.151372
*OR10J3*	up	8.073124	*C1orf159*	up	5.281698	*TRAF2*	up	7.866846
*GADD45A*	up	7.68622	*CFLAR*	up	5.159871	*C1orf159*	up	7.289466
*C1orf159*	up	5.831624	*GADD45A*	up	4.55888	*GRB2*	up	6.88594
*BCL2L11*	up	4.359662	*ATG3*	up	4.040016	*CASP1*	up	5.93121
*CASP6*	up	3.908813	*PARP2*	up	3.966246	*PARP2*	up	5.601994
*SQSTM1*	up	3.707083	*ATG7*	up	3.331992	*BMF*	up	5.39163
*CFLAR*	up	3.484138	*CASP1*	up	3.249445	*XIAP*	up	5.146085
*MAPK8*	up	3.117695	*MAPK8*	up	3.228049	*BCL2L1*	up	4.340562
*TRAF2*	up	3.097611	*EIF5B*	up	3.181103	*TNF*	up	4.008092
*CASP3*	up	2.563158	*DFFA*	up	2.584963	*ABL1*	up	3.989139
*DFFA*	up	2.395063	*SQSTM1*	up	2.313246	*GAA*	up	3.280956
*ATG7*	up	2.286881	*ACTB*	up	1.526069	*MCL1*	up	3.144046
*MCL1*	up	2.247928	*XIAP*	down	−1.23879	*CFLAR*	up	2.536053
*ATG3*	up	2.238787	*PVR*	down	−2.04264	*BECN1*	up	2.485427
*BCL2*	up	1.967169	*RAB25*	down	−2.16993	*DENND4A*	up	2.169925
*MAG*	up	1.891419	*PIK3C3*	down	−3.46597	*CASP6*	up	1.831877
*GAA*	up	1.807355	*ABL1*	down	−4.71644	*EIF5B*	up	1.495695
*CD40LG*	up	1.752749	*PARP1*	down	−5.40122	*PIK3C3*	up	1.344828
*BECN1*	up	1.589763	*ATG16L1*	down	−6.36107	*CTSB*	down	−2.0321
*DEFB1*	up	1.111031	*GALNT5*	down	−7.18378	*ULK1*	down	−2.83188
*XIAP*	down	−2.41414	*SYCP2*	down	−7.31442	*SPATA2*	down	−3.06178
*RAB25*	down	−2.69822	*SPATA2*	down	−10.957	*ATP6V1G2*	down	−3.64962
*PIK3C3*	down	−2.79286	*IGF1*	down	−11.894	*BCL2*	down	−3.97728
*FAS*	down	−3.4957				*COMMD4*	down	−5.99186
*TXNL4B*	down	−4.89579				*SYCP2*	down	−6.06953
*FOXI1*	down	−4.90593				*ATG16L1*	down	−6.34837
*ATG16L1*	down	−5.17632				*GALNT5*	down	−7.78973
*ABL1*	down	−5.2211						
*BMF*	down	−6.10476						
*PARP1*	down	−6.11582						
*APP*	down	−6.85723						
*AKT1*	down	−6.90857						
*COMMD4*	down	−8.98504						
*GALNT5*	down	−11.4963						

**Table 3:** DEGs in pathways of necroptosis, apoptosis, and autophagy.

Pathway	Genes
Necroptosis	*PARP1*; poly(ADP-ribose) polymerase 1
	*XIAP*; X-linked inhibitor of apoptosis
	*FAS*; Fas cell surface death receptor
	*MAPK8*; mitogen-activated protein kinase 8
	*BCL2*; BCL2 apoptosis regulator
	*TRAF2*; TNF receptor associated factor 2
	*CASP1*; caspase 1
	*CFLAR*; CASP8 and FADD like apoptosis regulator
	*SQSTM1*; sequestosome 1
	*SPATA2*; spermatogenesis associated 2
Apoptosis	*BCL2L11*; BCL2 like 11
	*PARP2*; poly(ADP-ribose) polymerase 2
	*PARP1*; poly(ADP-ribose) polymerase 1
	*GADD45A*; growth arrest and DNA damage inducible alpha
	*DFFA*; DNA fragmentation factor subunit alpha
	*AKT1*; AKT serine/threonine kinase 1
	*XIAP*; X-linked inhibitor of apoptosis
	*FAS*; Fas cell surface death receptor
	*MCL1*; MCL1 apoptosis regulator, BCL2 family member
	*MAPK8*; mitogen-activated protein kinase 8
	*BCL2*; BCL2 apoptosis regulator
	*BCL2L1*; BCL2 like 1
	*ACTB*; actin beta
	*TRAF2*; TNF receptor associated factor 2
	*CASP3*; caspase 3
	*CASP6*; caspase 6
	*CFLAR*; CASP8 and FADD like apoptosis regulator
Autophagy	*ATG7*; autophagy related 7
	*AKT1*; AKT serine/threonine kinase 1
	*PIK3C3*; phosphatidylinositol 3-kinase catalytic subunit type 3
	*ATG16L1*; autophagy related 16 like 1
	*MAPK8*; mitogen-activated protein kinase 8
	*BCL2*; BCL2 apoptosis regulator
	*BCL2L1*; BCL2 like 1
	*ATG3*; autophagy related 3
	*ULK1*; unc-51 like autophagy activating kinase 1
	BECN1; beclin 1
	*CFLAR*; CASP8 and FADD like apoptosis regulator
	*SQSTM1*; sequestosome 1

**Table 4:** Potential drug targets of menadione predicted by Pharmmapper database.

No.	Pharma Model	No. Feature	Fit	Norm Fit	Symbol
1	1ggt_A_cavity_2	5	2.48	0.496	*F13A1*
2	1x65_A_cavity_1	6	2.973	0.4954	*CSDE1*
3	1mj4_A_cavity_1	7	2.811	0.4016	*SUOX*
4	2f8x_C_cavity_3	7	2.687	0.3838	*NOTCH1*
5	2bug_A_cavity_1	8	2.881	0.3601	*HSP90AA1*
6	2p0k_A_cavity_2	9	2.684	0.2982	*SCMH1*
7	1o7k_C_cavity_1	10	2.652	0.2652	*NCF1*
8	1i0e_B_cavity_2	11	2.79	0.2536	*CKM*
9	1rc1_A_cavity_1	12	2.908	0.2423	*GART*
10	1ljr_A_cavity_1	12	2.868	0.239	*GSTT2B*
11	2pvs_B_cavity_2	12	2.355	0.1962	*PNLIPRP2*
12	3bpj_D_cavity_1	19	2.755	0.145	*EIF3J*
13	2vwi_B_cavity_1	26	2.816	0.1083	*OXSR1*
14	1pc2_A_cavity_1	26	2.795	0.1075	*FIS1*
15	1pu5_C_cavity_2	31	2.871	0.09262	*GM2A*
16	1us1_B_cavity_1	40	2.817	0.07042	*AOC3*
17	2k21_A_cavity_1	50	2.653	0.05306	*KCNE1*
18	2o36_A_cavity_2	70	2.919	0.04171	*THOP1*

**Table 5:** Drug targets of menadione predicted by Pharmmapper, Drugbank, and TTD.

Databases	Drug Targets
Pharmmapper	F13A1, CSDE1, SUOX, NOTCH1, HSP90AA1, SCMH1, NCF1, CKM, GART, GSTT2B, PNLIPRP2, EIF3J, OXSR1, FIS1, GM2A, AOC3, KCNE1, THOP1
Drugbank	GGCX, VKORC1, VKORC1L1, F2, F7, F9, F10, PROC, PROS1, PROZ, NQO2, NQO1, BGLAP,
TTD	AOX1, V1R

## Data Availability

Data presented in this study is contained within the article and supplementary material. Further inquiries can be directed to the corresponding author.

## Supplementary Materials

The following supporting information can be downloaded at: https://www.mdpi.com/article/10.3390/ph18081145/s1, Figure S1: Related to Figure 1. TNF-α (10 ng/mL) was administered to L929-FADD-KO cells independently or combined with Nec-1 (30 μM); Figure S2: Related to Figure 2. (A) Smac mimetics and Z-VAD-FMK were administered to HT-29 cells independently or combined. Cell viability was assessed after 24 h (n = 3). (B) TNF-α was administered to HT-29 independently or combined with Smac mimetics and Z-VAD-FMK. Cell viability was assessed after 24 h (n = 3); Figure S3: Related to Figure 3. Menadione (3 μM and 8 μM) was administered to HEK293T, SW620 and HCT116 cells and cell viability was assessed (n = 3); Figure S4: Related to Figure 4. The pathways from the KEGG and GO results were analyzed using the Enrichment Map plugin in Cytoscape. The node color represents the number of enriched genes in each pathway, with darker colors indicating a higher number of genes; Figure S5: Related to Figure 5. (A–C) display the results of KEGG Mapper, showing pathway diagrams for necroptosis (A), apoptosis (B) and autophagy (C); Figure S6: Related to Figure 5. (A) PPI network of DEGs in menadione groups. STRING database was used to for PPI analysis of 47 DEGs in menadione groups, and the PPI score was set as >0.400; Figure S7: Related to Figure 5. Involvement of MAPK8 in (A) KEGG and (B) GO enrichment pathways; Figure S8: Related to Figure 7. The pharmacophore structural models of menadione drug targets; Table S1: Cell viability (%) of L929-FADD-KO cells after treating with 12 candidate drugs; Table S2: Cell viability (%) of L929-FADD-KO cells after treating with candidate drugs and Nec-1; Table S3: Cell viability (%) of wild-type L929 cells after treating with candidate drugs and Nec-1.

## Abbreviations

The following abbreviations are used in this manuscript:

CRC	Colorectal cancer
PCD	Programmed cell death
TNF-α	Tumor necrosis factor alpha
TSZ	TNF-α, Smac mimetics and Z-VAD-FMK
DEG	Differentially expressed gene
GSEA	Gene set enrichment analysis
PPI	Protein–protein interaction
GO	Gene ontology
KEGG	Kyoto encyclopedia of genes and genomes
TTD	Therapeutic target database
TNFR1	Tumor necrosis factor receptor 1
NF-κB	Nuclear factor kappa B
RIA	RIPK1-independent apoptosis
RDA	RIPK1-dependent apoptosis
TRADD	TNFR1-associated death domain protein
FADD	Fas-associated via death domain
