# Supplementary material for: Screening of an FDA-Approved Drug Library: Menadione Induces Multiple Forms of Programmed Cell Death in Colorectal Cancer Cells via MAPK8 Cascades
Source: Pharmaceuticals (Basel). 2025 Jul 31;18(8):1145. doi: 10.3390/ph18081145 (PMC12389538; doi:10.3390/ph18081145)
Supplement: Supplementary file 1 [file pharmaceuticals-18-01145-s001.zip › pharmaceuticals-3743930-supplementary.pdf]

Supplementary Tables

Supplementary Table S1. Cell viability (%) of L929-FADD-KO cells after treating with 12 candidate drugs.

Drug	Well 1	Well 2	Well 3
Menadione	4.31	3.5	3.91
Disulfira	43.78	32.11	36.16
Bosutinib	36.04	28.43	24.21
Evans blue	53.35	49.26	39.09
Crystal violet	20.65	18.44	16.33
Dronedarone hydrochloride	19.14	22.57	31.15
Crizotinib	0.65	0.41	0.56
Bazedoxifene acetate	32.38	31.45	38.98
Vandetanib	38.82	38.92	37.33
Vortioxetine HBr	1.84	1.86	1.7
Fingolimodhydrochloride	9.46	13.87	10.37
Mesylate	8.47	3.79	6.31

Supplementary Table S2. Cell viability (%) of L929-FADD-KO cells after treating with candidate drugs and Nec-1.

Drugs	Drug			Drug + Nec-1		
	Well 1	Well 2	Well 3	Well 1	Well 2	Well 3
Menadione	29.69	24.75	31.47	38.52	42.32	34.27
Disulfira	43.66	36.48	45.94	42.48	33.02	38.48
Bosutinib	40.88	41.41	37.68	36.48	32.89	26.76
Evans blue	36.91	35.34	39.63	36.7	40.07	42.13
Crystal violet	14.59	17.31	15.74	17.53	18.91	19.85
Dronedarone hydrochloride	47.64	58.23	51.79	44.19	44.76	44.94
Crizotinib	18.74	16.31	25.04	12.72	17.23	17.38
Bazedoxifene acetate	51.22	58.8	54.36	59.36	58.24	65.73
Vandetanib	63.95	69.24	67.38	66.67	65.92	62.92
Vortioxetine HBr	13.78	17.45	24.03	14.66	16.76	18.54
Fingolimodhydrochloride	30.76	28.33	38.05	22.1	16.95	18.71
Mesylate	51.5	50.64	55.79	42.7	45.51	44.01

Supplementary Table S3. Cell viability (%) of wild-type L929 cells after treating with candidate drugs and Nec-1

Drugs	Drug			Drug + Nec-1		
	**Well 1**	**Well 2**	**Well 3**	**Well 1**	**Well 2**	**Well 3**
Menadione	29.56	33	31.57	55.2	56.26	52.03
Disulfira	22.79	20.74	24.41	22.06	18.97	20.59
Bosutinib	36.91	36.03	30.15	39.26	37.21	33.97
Evans blue	30.3	35.84	33.23	38.34	35.05	37.29
Crystal Violet	7.43	8.08	6.92	17.26	16.73	17.39
Dronedarone hydrochloride	30.67	29.68	31.16	36.1	37.65	33.23
Crizotinib	0.53	0.54	0.44	3.11	2.06	2.13
Bazedoxifene acetate	26.72	30.05	26.6	22.4	23.29	29.53
Vandetanib	25.74	25.62	26.48	37.96	28.72	28.06
Vortioxetine HBr	0.7	0.59	0.51	1.32	1.34	1.9
Fingolimodhydrochloride	3.37	3.26	2.7	3.52	1.7	1.34
mesylate	5.55	7.38	4.01	42.82	37.15	32.27

Supplementary Figures

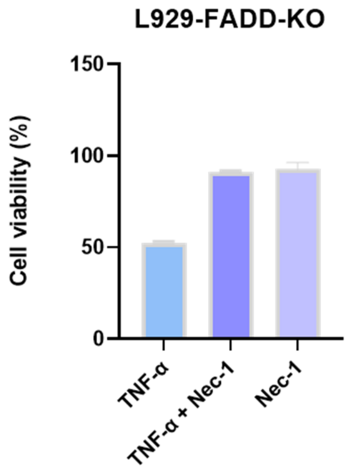

Supplementary Figure S1. Related to Figure 1.

TNF-*α* (10 ng/mL) was administered to L929-FADD-KO cells independently or combined with Nec-1 (30 μM). Cell viability was assessed after 24 h (n = 3).

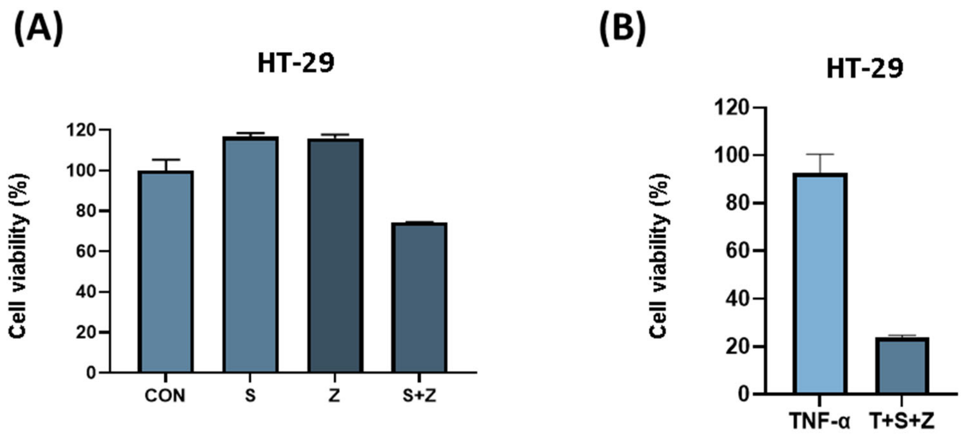

Supplementary Figure S2. Related to Figure 2.

(**A**) Smac mimetics and Z-VAD-FMK were administered to HT-29 cells independently or combined. Cell viability was assessed after 24 h (n = 3). (**B**) TNF-*α* was administered to HT-29 independently or combined with Smac mimetics and Z-VAD-FMK. Cell viability was assessed after 24 h (n = 3). S: Smac mimetics, 100 nM; Z: Z-VAD-FMK,20 μM. Data of cell viability are presented as mean ± SEM (n = 3).

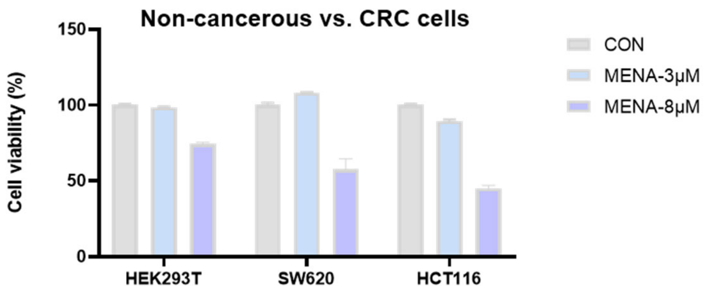

Supplementary Figure S3. Related to Figure 3.

Menadione (3 μM and 8 μM) was administered to HEK293T, SW620 and HCT116 cells and cell viability was assessed (n = 3). Data of cell viability are presented as mean ± SEM (n = 3).

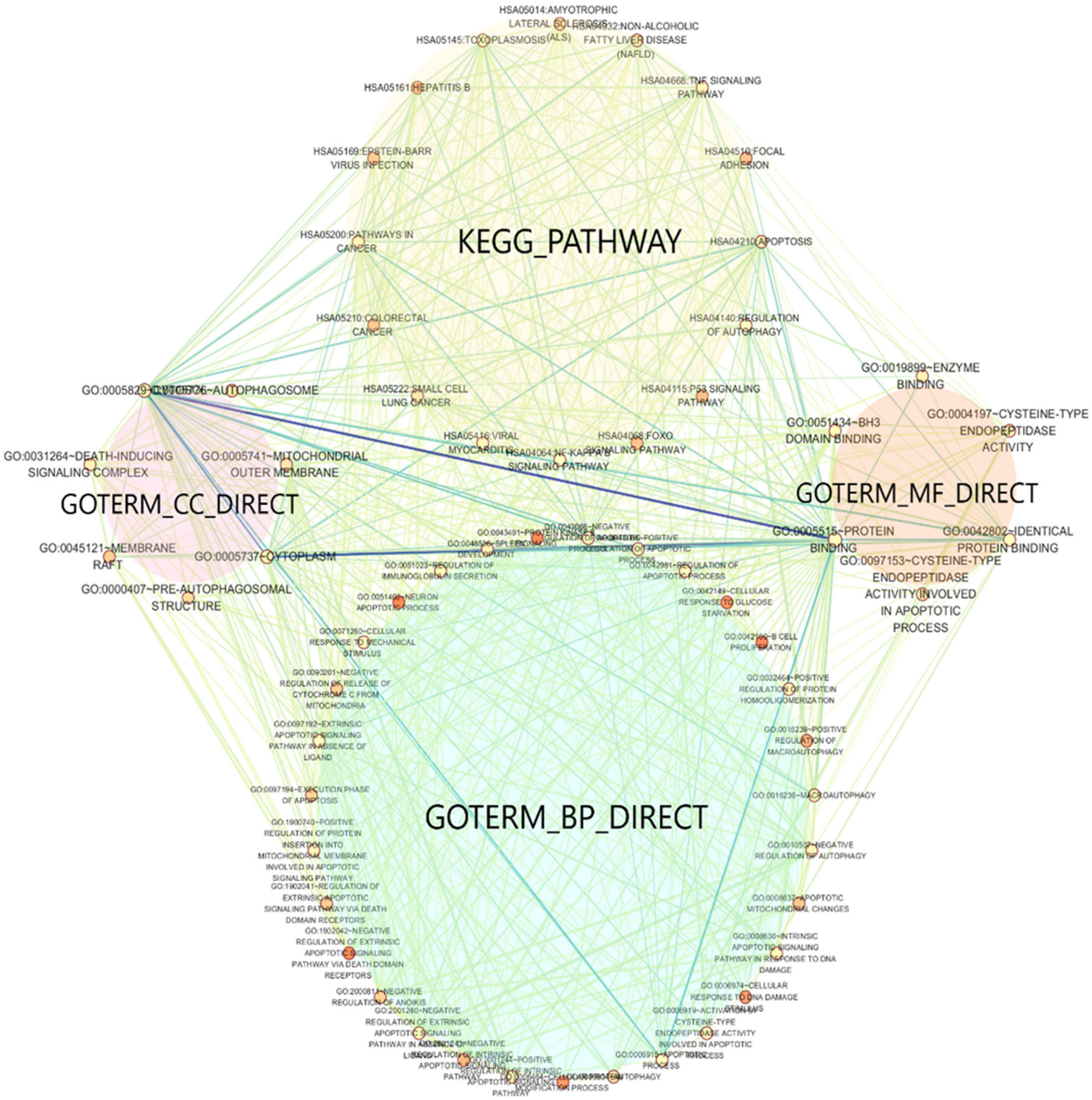

Supplementary Figure S4. Related to Figure 4.

The pathways from the KEGG and GO results were analyzed using the Enrichment Map plugin in Cytoscape. The node color represents the number of enriched genes in each pathway, with darker colors indicating a higher number of genes. The edge color represents the overlap size, which is the number of shared genes between two pathways, with darker colors indicating a larger overlap. The edge thickness represents the similarity coefficient, with thicker lines indicating a higher similarity coefficient.

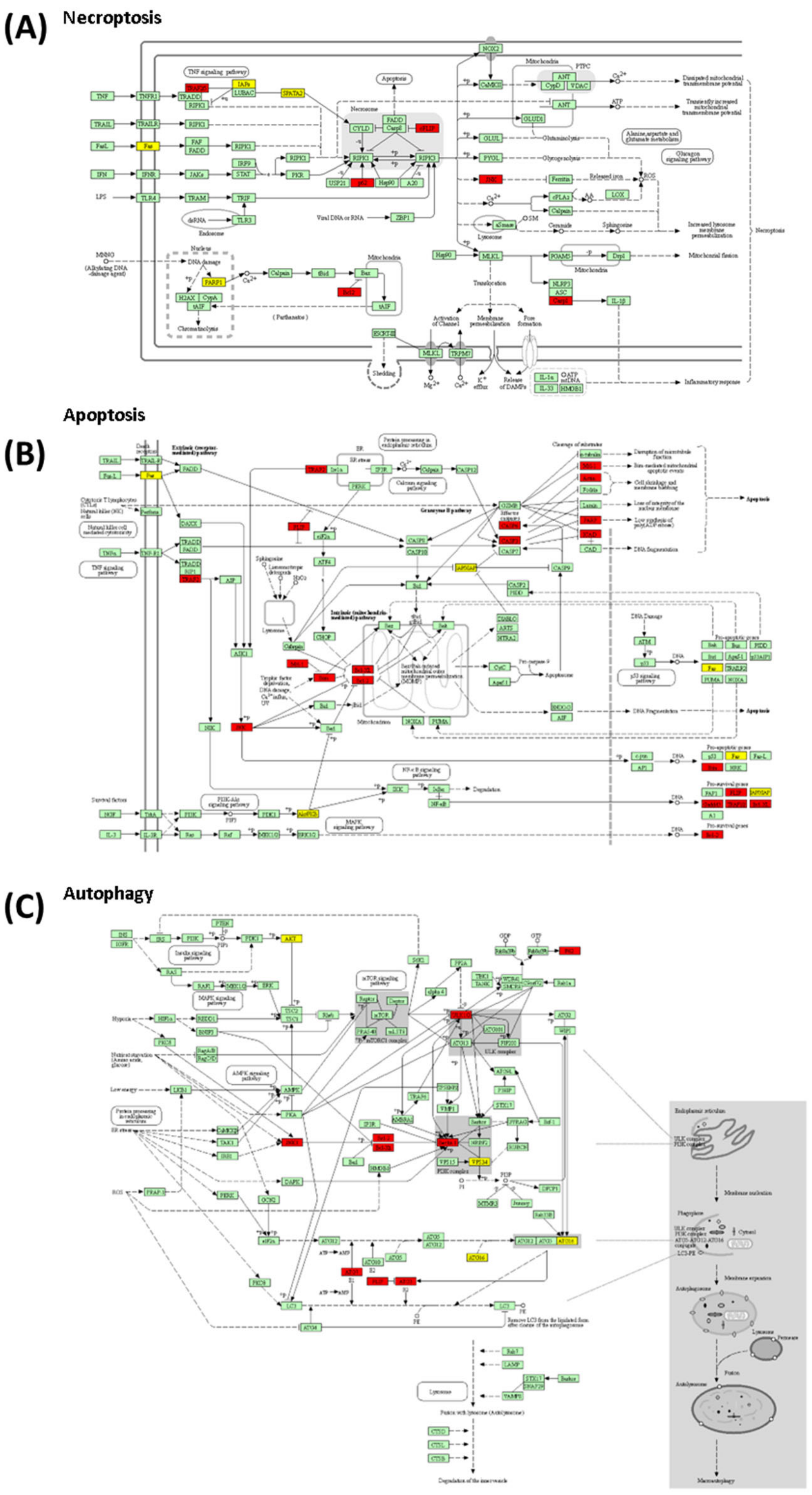

Supplementary Figure S5. Related to Figure 5.

(**A–C**) display the results of KEGG Mapper, showing pathway diagrams for necroptosis (**A**), apoptosis (**B**) and autophagy (**C**). The nodes in red represent up-regulated DEGs of menadione groups, and nodes in yellow indicate down-regulated DEGs. Overall, the diagrams of KEGG Mapper show the intricate interplay among various molecular components and signaling pathways governing necroptosis, apoptosis and autophagy.

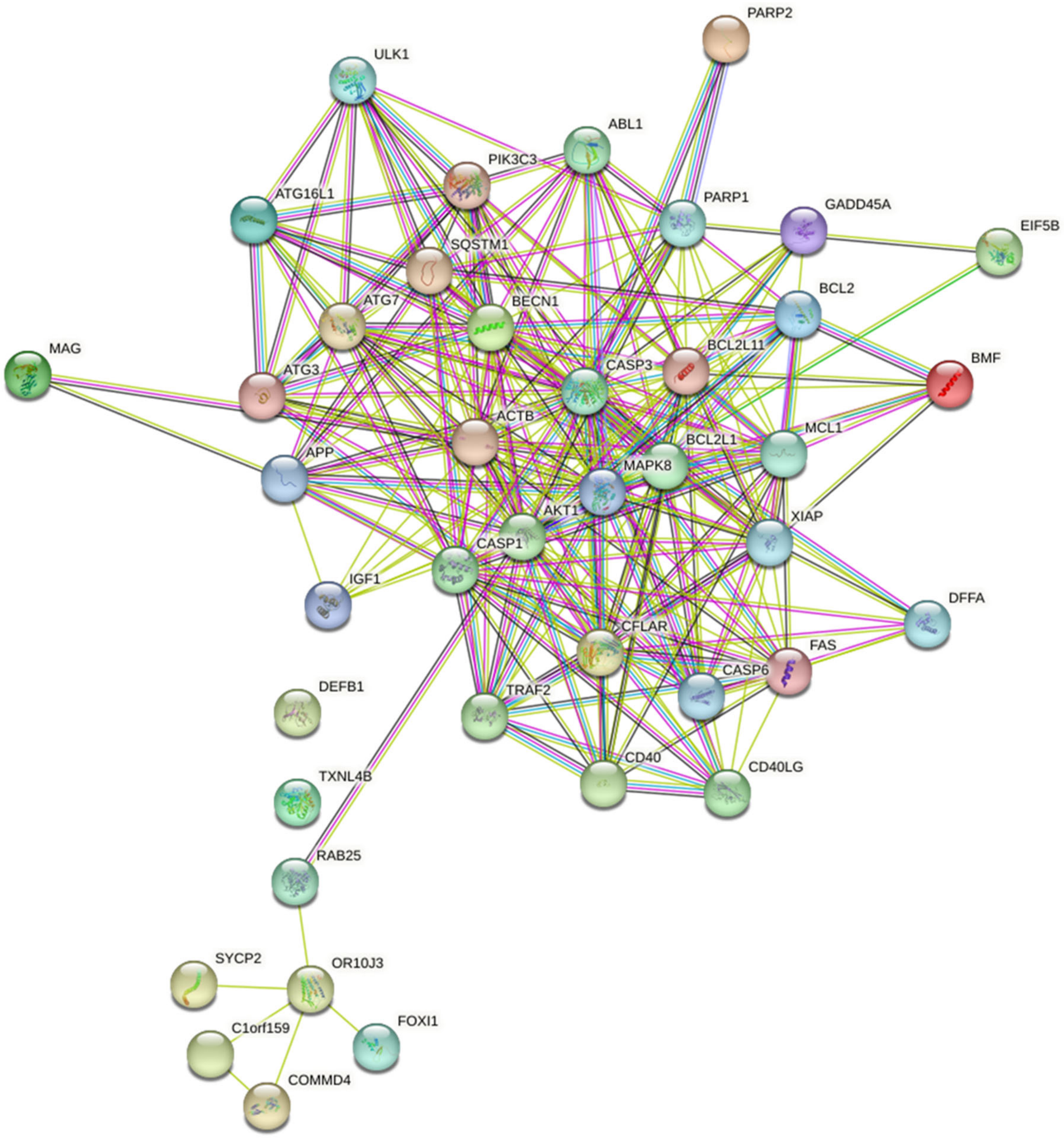

Supplementary Figure S6. Related to Figure 5.

PPI network of DEGs in menadione groups. STRING database was used to for PPI analysis of 47 DEGs in menadione groups, and the PPI score was set as >0.400.

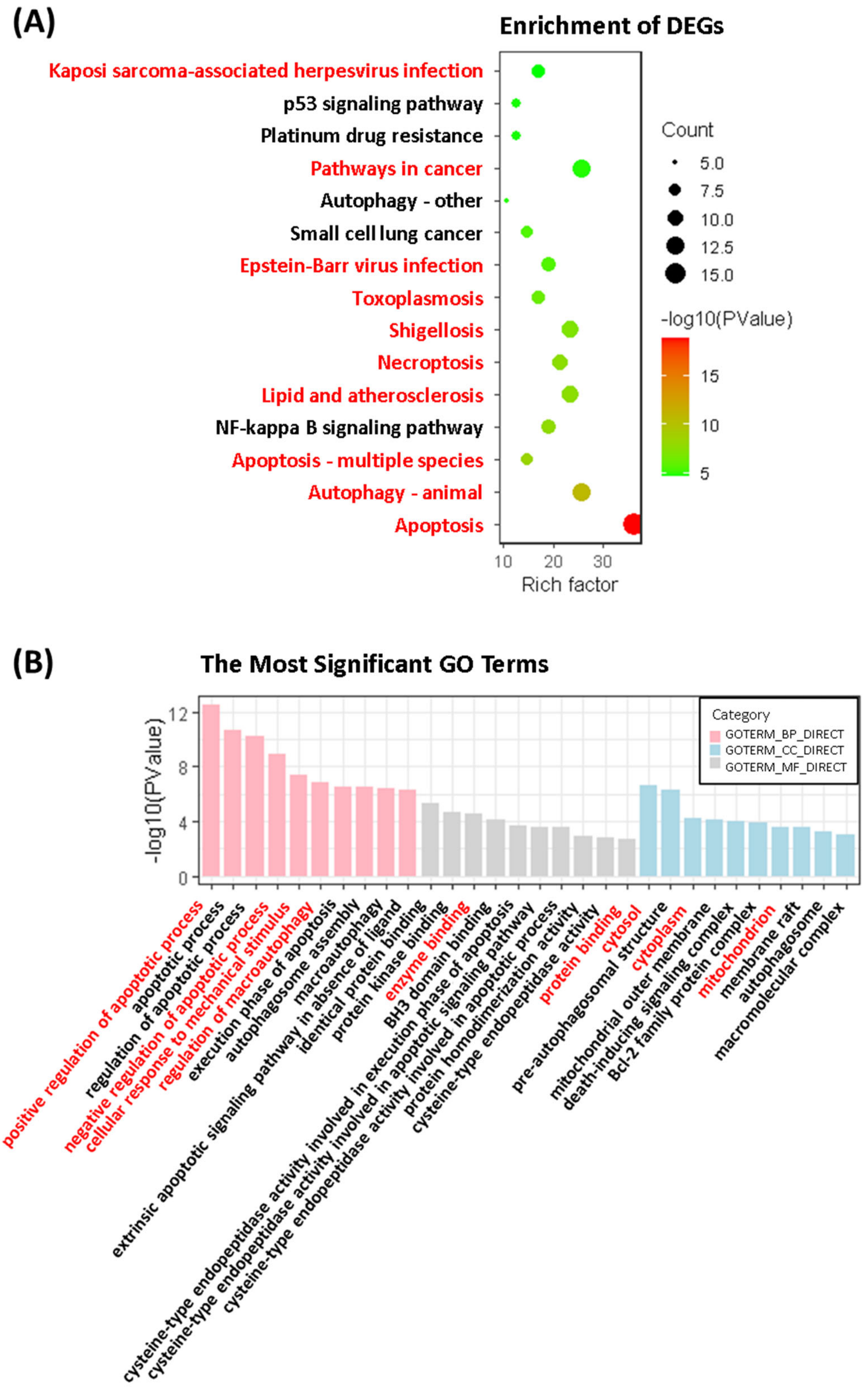

Supplementary Figure S7. Related to Figure 5.

Involvement of MAPK8 in (**A**) KEGG and (**B**) GO enrichment pathways. The pathways highlighted in red indicate involvement of MAPK8.

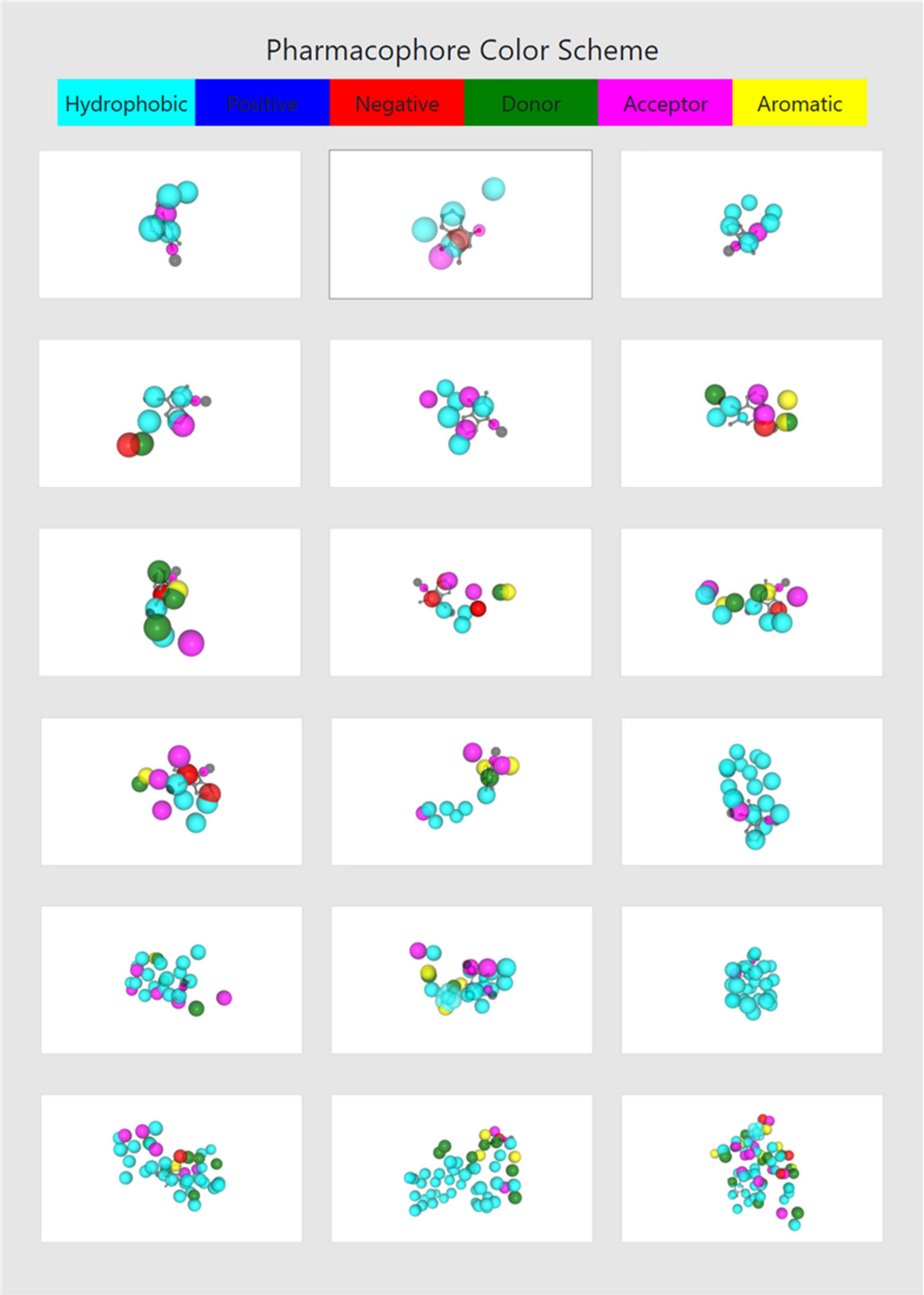

Supplementary Figure S8. Related to Figure 7.

The pharmacophore structural models of menadione drug targets. The Pharmmapper database was utilized to predict the potential drug targets of menadione, resulting in a total of 18 identified drug targets.
